# Supplementary material for: Cyclosporin A Increases Mitochondrial Buffering of Calcium: An Additional Mechanism in Delaying Mitochondrial Permeability Transition Pore Opening
Source: Cells. 2019 Sep 7;8(9):1052. doi: 10.3390/cells8091052 (PMC6770067; doi:10.3390/cells8091052)
Supplement: Supplementary file 1 [file cells-08-01052-s001.pdf]

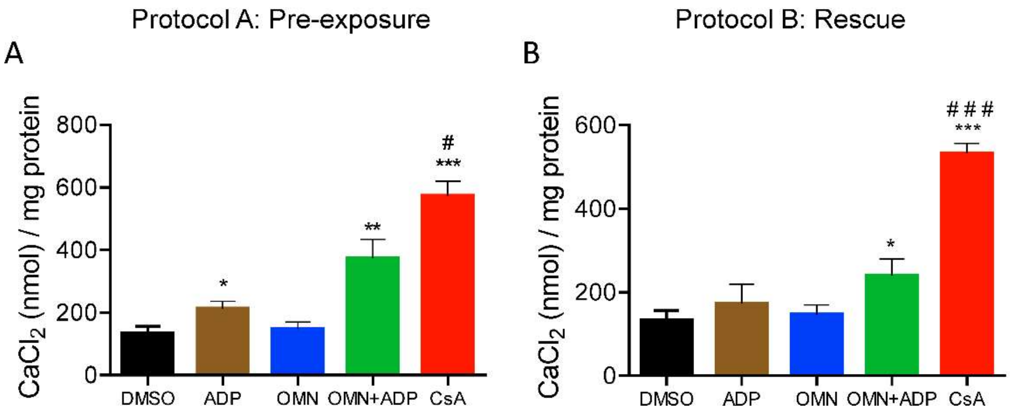

Figure S1: Quantification of calcium retention capacity (CRC) for each treatment, DMSO (control), ADP, oligomycin (OMN), OMN+ADP, and CsA during Protocol A (A) and Protocol B (B). The amount of Ca^{2+} was normalized to the mitochondrial content (mg protein). All values are mean ± SEM. ($^{*}p < 0.05$; $^{**}\ p < 0.01$; $^{***}\ p < 0.001$ between specified treatment vs. DMSO, $^{\#}\ p < 0.05$; $^{\#\#\#}\ p < 0.001$ OMN+ADP vs. CsA). Each experiment was repeated three to five times.

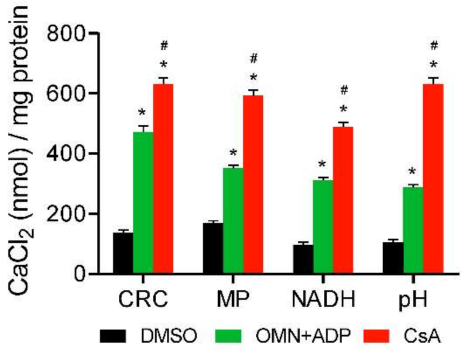

Figure S2: Average Ca^{2+} added before mPTP opening, $\Delta\Psi_m$ collapse, NADH oxidation, and matrix acidification. The amount of Ca^{2+} was normalized to the mitochondrial content (mg protein). All values are mean ± SEM. $^{*}\ p < 0.001$ (CsA vs. DMSO) and $^{\#}\ p < 0.001$ (OMN+ADP vs. DMSO). CRC = calcium retention capacity, MP = membrane potential ($\Delta\Psi_m$). Each experiment was repeated three to five times.

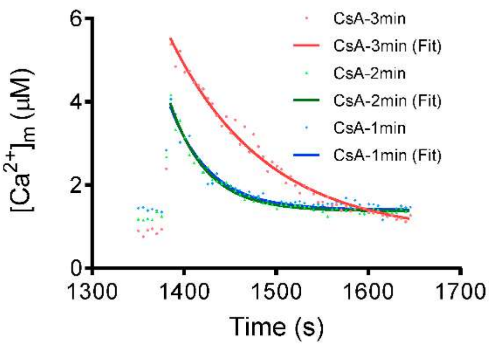

Figure S3: Trend-fits for calculation of buffering rate. Average data traces from three to five experiments (represented in Figure 6B (right panel, box) showing the effect of CsA on matrix buffering rate at 1, 2, and 3 min after free $[Ca^{2+}]_m$ increase) were fit with a single exponential, $y(t) = A_1 * \exp(-t/t_1) + y_0$, where t_1 represents the time

to achieve ss[Ca^{2+}]$_m$ after CsA application. Decay time constants were 43, 40, and 105 ms, respectively, for application of CsA at 1, 2, and 3 min after increase in free [Ca^{2+}]$_m$.

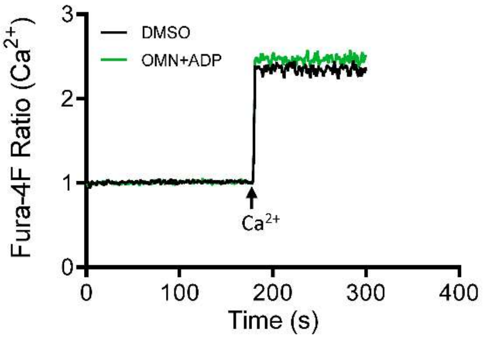

Figure S4: Fura-4F ratio representing the change in [Ca^{2+}]$_e$ before and after adding a 20 μM $CaCl_2$ bolus in the absence or presence of 250 μM ADP in the mitochondria-free experimental buffer. Note that the increase in Ca^{2+} fluorescence signal intensity (Fura-4F ratio) when 250 μM ADP was added was similar to that observed in the presence of water (control).

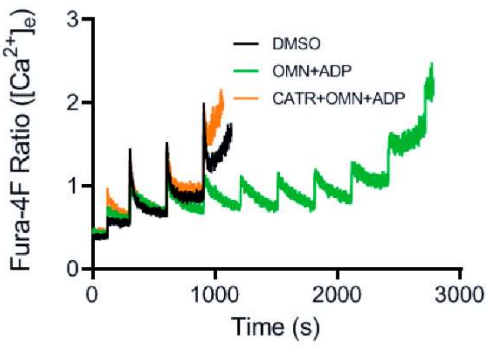

Figure S5: Representative raw traces of extra-matrix Ca^{2+} fluorescence (Fura-4F ratio) and Ca^{2+} uptake in mitochondria pretreated with 0.5% DMSO (control), OMN+ADP, and CATR (carboxyatractyloside)+OMN+ADP prior to mPTP opening. CATR had no added effect on Ca^{2+} uptake over DMSO.
